# Early Postoperative Volume Overload is a Predictor of 1-Year Post-Transplant Mortality in Pediatric Heart Transplant Recipients

**DOI:** 10.1007/s00246-023-03134-9

**Published:** 2023-03-22

**Authors:** Michelle J. Lim, Myung-Shin Sim, Sylvia Pan, Juan Alejos, Myke Federman

**Affiliations:** 1 Division of Critical Care, Department of Pediatrics, UC Davis School of Medicine, UC Davis Children’s Hospital, 2516 Stockton Boulevard, Sacramento, CA USA; 2 Department of General Internal Medicine, Statistics Core, UCLA Geffen School of Medicine, Los Angeles, CA USA; 3 Division of Cardiology, Department of Pediatrics, UCLA Geffen School of Medicine, Mattel Children’s Hospital, Los Angeles, CA USA; 4 Division of Critical Care, Department of Pediatrics, UCLA Geffen School of Medicine, Mattel Children’s Hospital, Los Angeles, CA USA

**Keywords:** Pediatric heart transplant, Fluid overload, Cardiac function, Cardiac transplantation, Rejection, Mortality

## Abstract

Fluid restriction and diuretic management are mainstays in the postoperative management of cardiac patients, at risk of volume overload and its deleterious effects on primary cardiac function and multi-organ systems. The importance of fluid homeostasis is further emphasized among orthotopic heart transplant recipients (OHT). We sought to investigate the relationship between postoperative volume overload, mortality, and allograft dysfunction among pediatric OHT recipients within 1-year of transplantation. This is a retrospective cohort study from a single pediatric OHT center. Children under 21 years undergoing cardiac transplantation between 2010 and 2018 were included. Cumulative fluid overload (cFO) was assessed as percent fluid accumulation adjusted for preoperative body weight. Greater than 10% cFO defined those with postoperative cFO and a comparison of postoperative cFO vs. no postoperative cFO (< 5%) is reported. 102 pediatric OHT recipients were included. Early cFO at 72 h post-OHT occurred in 14% and overall cFO at 1-week post-OHT occurred in 23% of patients. Risk factors for cFO included younger age, lower weight, and postoperative ECMO. Early cFO was associated with postoperative mortality at 1-year, OR 8.6 (95% CI 1.4, 51.6), *p* = 0.04, independent of age and weight. There was no significant relationship between cFO and allograft dysfunction, measured by rates of clinical rejection and cardiopulmonary filling pressures within 1-year of transplant. Early postoperative volume overload is prevalent and associated with increased risk of death at 1-year among pediatric OHT recipients. It may be an important postoperative marker of transplant survival, and this relationship warrants further clinical investigation.

## Introduction

Fluid therapy is widely recognized as essential practice for the acute resuscitation of critically ill children [1, 2]. However, aggressive fluid resuscitation beyond the acute resuscitation period may lead to early fluid overload and worse clinical outcomes [3]. Early fluid overload has been further investigated both as an early prognostic marker of poor clinical outcomes and an important potential modifiable risk factor in pediatric critical illness, shaping current clinical standards of fluid-goal directed therapy [3–11].

Post-cardiac surgery patients are considered particularly vulnerable to both postoperative volume overload and its secondary complications that result in severe morbidity and mortality [12, 13]. The risk of volume overload is multifactorial and often compounded by the effect of preoperative heart failure, the physiological changes following cardiopulmonary bypass that include capillary leak and low cardiac output syndrome, and renal dysfunction [14–18]. Further, early fluid overload following postoperative low cardiac output syndrome reduces end-organ perfusion pressure and propagates a cycle of dysregulated pathophysiological mechanisms that exacerbate systemic organ failure, tissue edema, and volume overload [19]. Fluid restriction and aggressive diuretic support have been the mainstay of post-operative management of cardiac surgical patients, among adult and pediatric patients [12, 13, 20, 21].

In orthotopic heart transplant recipients (OHT), the importance of fluid homeostasis is further emphasized in clinical practice. A maladaptive neuroendocrine and impaired renal response to plasma volume expansion have been observed in postoperative adult OHT patients and associated with both fluid overload and cardiac dysfunction in the early postoperative period [22]. Although the causality of this relationship is not entirely clear, interruptions to key neural and humoral homeostatic mechanisms from graft denervation has been previously described [23, 24]. Among sparse pediatric literature, a recent small retrospective study reported a high incidence of postoperative volume overload among pediatric heart transplant recipients and an independent association with poor short-term outcomes including postoperative mortality in those with severe fluid overload [21].

In our study, we sought to describe the incidence of postoperative cumulative fluid overload (cFO) in a pediatric OHT population and the perioperative risk factors associated with fluid overload. Given a growing body of pediatric cardiac surgical literature reporting the association of both postoperative peak and cumulative fluid overload at 72 h and poor outcomes [13, 25], we assessed cFO across two time points: (1) early (at 72 h of transplantation) and (2) overall (at 1-week post-transplantation). Second, we aimed to explore the relationship between cFO and early and late post-transplant outcomes assessed within the first year of transplantation. Specifically, we investigated the relationship between cFO and allograft dysfunction within the first year of transplant. We hypothesized cFO to be associated with allograft dysfunction as measured by rates of empiric clinical rejection, graft loss/death, and elevated cardiopulmonary filling pressures/ventricular strain observed on routine cardiac catheterization within the first year of transplant.

## Patients and Methods

### Setting and Study Design

Children who were between the ages of 0 and 21 years of age undergoing cardiac transplantation between January 2010 and May 2018 were included in our analysis. This was a retrospective cohort study from a single-center large volume pediatric heart transplant center in a large metropolitan city, UCLA Mattel Children’s Hospital. The study was approved by the Institutional Review Board at the University of California, Los Angeles. There were no patients who underwent more than one cardiac transplantation during the time period and all indications of transplant, including repeat transplantation for graft failure, were included in the study.

### Data Collection

A retrospective chart review was performed with demographic, preoperative, intraoperative, and post-operative variables that were all identified prior to data collection initiation. Data was obtained from electronic medical record. Demographic variables included age, gender, and race. Additional preoperative variables included were last recorded preoperative weight and baseline serum creatinine (both measured up until 1 week prior to transplantation), presence of a cyanotic heart lesion, primary indication for cardiac transplantation, and the presence of mechanical circulatory support at time of transplantation. Perioperative variables included cardiopulmonary bypass time (CPB), aortic cross-clamp time, and donor ischemic time, and need for immediate mechanical circulatory support (MCS) due to primary graft failure following transplantation. All patients with postoperative graft failure needing MCS support was due to the inability to separate initially from cardiopulmonary bypass with primary transplantation. There were no patients needing late MCS support with the initial postoperative recovery. Vasoactive inotrope score (VIS) was calculated using variables recorded within the first 72 h of transplantation. The VIS equation used for the calculation was the following: (dopamine + dobutamine) + (10 × milrinone) + (100 x (epinephrine + norepinephrine + phenylephrine)) in units of mcg/kg/min [26].

Percent fluid accumulation was grouped into three categories, cumulative fluid balance (cFO) of < 5%, 5–10%, and > 10%, respectively. Percent fluid calculation was calculated using accepted standards of fluid overload classification, using the following equation: (total volume input—volume of fluid output)/admission weight (kg) × 100 [27]. Early fluid overload was defined as patients with cumulative fluid overload of > 10% at 72 h of transplantation and overall postoperative cumulative fluid overload was defined as > 10% at 7 days following transplantation. If a patient was discharged or died prior to 7 days of transplantation, the cumulative fluid balance calculated up until day of discharge was used for analysis.

### Outcome Measures

Early postoperative outcomes included postoperative mortality, development of acute kidney injury (AKI) within the first week of transplantation, and the use of continuous renal replacement therapy (CRRT) during the initial postoperative hospitalization stay. Postoperative mortality was defined as death related to any-cause during the initial hospitalized postoperative period following cardiac transplantation. Acute kidney injury was defined by the KDIGO definition of acute AKI, stages 1–3 with both serum creatinine and urine output collected daily within the first 7 days of postoperative stay [28]. Baseline preoperative creatinine levels were collected for comparison. Renal failure was defined as those with KDIGO stage 3 AKI.

Longer term outcomes were limited to data collection within the first year of cardiac transplantation as all post-transplant follow-up which included routine cardiac catheterization surveillance and any hospitalizations were exclusively followed within the UCLA health system during this time period. Clinical rejection was defined as new onset heart failure or left ventricular systolic dysfunction that warranted empiric treatment for acute rejection within the first year of transplantation [29], irrespective of corresponding biopsy results. Severe clinical rejection was defined as treatment for acute rejection and the presence of hemodynamic compromise, requiring the use of inotropic and/or vasopressor use, initial IV fluid resuscitation, and/or mechanical circulatory support with the use of extracorporeal life support (ECLS) or ventricular assist device (VAD).

Routine cardiac catheterization surveillance occurred at strict routine time points following transplantation at 1–4 weeks, 3–6 months, and 1-year following initial transplantation. If more than one cardiac catheterization procedure occurred within the aforementioned time frame, the worst hemodynamic data from a single cardiac catheterization procedure, as measured by cardiopulmonary filling pressures, was collected. Data collection from cardiac catheterization was limited to the collection of right atrial pressure (RAP), pulmonary capillary wedge pressure (PCWP), and cardiac index (CI) at the time of the cardiac catheterization procedure.

### Statistical Analysis

Student *t*-test and Chi-square test have been used in comparing continuous and categorical variables, respectively, between those with no fluid accumulation, (cFO) < 5% vs. cFO > 10% groups. Logistic regression models have been used for binary outcomes incorporating cFO group as a main study variable adjusting for age at transplant and admission weight. Binary outcomes we analyzed are mortality, acute kidney injury, rejections (clinical rejection, clinical or biopsy proven rejection, and severe clinical rejection), right atrial pressure (RAP) > 8 mmHg, pulmonary capillary wedge pressure (PCWP) > 12 mmHg, and low cardiac index (CI) as measured by a CI < 2 L/min/m^2^. The cFO (< 5% vs 10%) evaluated at 72 h and at 1 week were analyzed separately in all models. The frequency of hospitalizations within the first year of cardiac transplantation has been analyzed using general linear model with negative binomial link. Length of hospitalization was analyzed using general linear model. All tests were 2-sided and *p*-value ≤ 0.05 was considered statistically significant. SAS 9.4 (Cary, NC) has been used for all analyses.

## Results

### Risk Factors for Fluid Overload

A comparison between baseline characteristics of those with early versus overall cFO, are presented in Tables 1 and 2, respectively. A total of 102 patients met study criteria for analysis during the time period. Early cFO occurred in 14% (14/102) of total cardiac transplant recipients and overall cFO occurred in 23% (23/102). Both time points for fluid overload showed similar findings. Overall postoperative cFO was most likely to occur in younger patients, with 60.9% (14/23) of patients with postoperative fluid overload (*p* < 0.001) were less than 1 year of age at time of transplantation compared to 12.3% (8/65) of patients without volume overload (< 5%). Corresponding to age, a lower preoperative weight (early, *p* = 0.01; overall, *p* < 0.001) was noted to be associated with fluid overload both within the early postoperative period and overall, at 1-week post-transplant. The presence of a cyanotic heart lesion (*p* = 0.05) was also noted to be a significant risk factor for volume overload in the early postoperative period.

**Table 1:** Baseline characteristics, early cFO (at 72 h)

Demographics	cFO < 5% (*N* = 59)	cFO > 10% (*N* = 14)	*P*-value
Age at OHT, years (mean, sd)	9.9 (7.0)	5.3 (6.2)	**0.03**
Infant < 1 year at OHT	12 (20.3%)	4 (28.6%)	0.5
Gender			
Male	30 (50.9%)	6 (42.9%)	0.59
Female	29 (49.2%)	8 (57.1%)	
Race			
Hispanic	33 (55.9%)	8 (57.1%)	0.94
Weight, kg (mean, sd)	38.9 (27.7)	19.4 (19.4)	**0.01**
Cyanotic lesion	14 (23.7%)	7 (50%)	**0.05**
Indications for transplant			
Congenital	12 (20.3%)	7 (50%)	0.08
Myocarditis	2 (3.4%)	1 (7.1%)	
Cardiomyopathy	38 (64.4%)	4 (28.6%)	
Re-transplant failure	7 (11.9%)	2 (14.3%)	
Support at transplant			
VAD	14 (23.7%)	2 (14.3%)	0.44
ECLS	5 (8.5%)	0 (0%)	0.26
Vasoactives	8 (13.6%)	1 (7.1%)	0.51
Peri/post-transplant			
Cardiopulmonary bypass (min) (mean, sd)	113.7 (59.8)	108.5 (31.5)	0.75
Aortic cross-clamp (min) (mean, sd)	68.7 (33.0)	68.1 (31.5)	0.95
Donor ischemic (min) (mean, sd)	194.3 (90.7)	199.5 (122.0)	0.87
Max VIS 72H (mean, sd)	12.0 (5.2)	17.4 (13.5)	0.25
Graft failure/postoperative ECLS	3 (5.1%)	4 (28.6%)	**0.01**

Bold values indicate statistical significance (*p* ≤ 0.05)

Baseline perioperative characteristics of patients with early postoperative cumulative fluid overload (cFO) assessed as cumulative fluid balance up to 72 h following post-cardiac transplantation, compared to those without postoperative cFO at given time period

*OHT* orthotopic heart transplant, *VAD* ventricular assist device, *ECLS* extracorporeal life support, *VIS* vasoactive inotrope score at 72 h following cardiac transplantation

**Table 2:** Baseline characteristics, overall cFO (at 1 week)

Demographics	cFO < 5% (*N* = 65)	cFO > 10% (*N* = 23)	*P*-value
Age at OHT, yrs (mean, sd)	11.9 (6.4)	1.7 (2.3)	** < 0.001**
Infant < 1 year at OHT	8 (12.3%)	14 (60.9%)	** < 0.001**
Gender			
Male	30 (46.2%)	10 (43.5%)	0.83
Female	35 (53.9%)	13 (56.5%)	
Race			
Hispanic	35 (53.9%)	17 (73.9%)	0.09
Weight, kg (mean, sd)	44.4 (25.3)	9.3 (5.6)	** < 0.001**
Cyanotic lesion	15 (23.1%)	8 (34.5%)	0.27
Indications for transplant			
Congenital	10 (15.4%)	8 (34.8%)	**0.03**
Myocarditis	2 (3.1%)	2 (8.7%)	
Cardiomyopathy	40 (61.5%)	13 (56.5%)	
Re-transplant failure	13 (20%)	0 (0%)	
Support at transplant			
VAD	16 (24.6%)	3 (13.0%)	0.25
ECLS	5 (7.7%)	1 (4.4%)	0.58
Vasoactives	7 (10.8%)	4 (17.4%)	0.41
Peri/post-transplant			
Cardiopulmonary bypass (min) (mean, sd)	114.7 (58.7)	105.0 (28.9)	0.46
Aortic cross-clamp (min) (mean, sd)	70.5 (31.4)	59.3 (24.5)	0.12
Donor ischemic (min) (mean, sd)	188.7 (85.6)	221.1 (110.5)	0.18
Max VIS 72H (mean, sd)	12.1 (5.4)	15.1 (11.3)	0.42
Graft failure/postoperative ECLS	3 (4.6%)	4 (17.4%)	**0.05**

Bold values indicate statistical significance (*p* ≤ 0.05)

Baseline perioperative characteristics of patients with overall postoperative cumulative fluid overload (cFO) assessed as cumulative fluid balance up to 1-week following post-cardiac transplantation, compared to those without postoperative fluid overload without postoperative cFO at given time period

*OHT* orthotopic heart transplant, *VAD* ventricular assist device, *ECLS* extracorporeal life support, *VIS* vasoactive inotrope score at 72 h following cardiac transplantation

There was no significant difference in the proportion of patients who required preoperative ECMO or VAD, or higher mean times for cardiopulmonary bypass or donor ischemic times among patients with early or overall postoperative volume overload compared to those without volume overload. Patients, however, who had immediate graft failure and required the use of postoperative ECMO, were noted to have higher incidence of both early and overall postoperative volume overload (early, *p* = 0.01; overall, *p* = 0.05) (Tables 1 and 2).

### Fluid Overload and Early Clinical Outcomes

Both early and overall cFO was not significantly associated with in-hospital mortality (Table 3). Further, there was no significant relationship between early and overall cFO with acute kidney injury (early cFO, OR 1.3 [95% CI 0.4, 4.1], *p* = 0.69; overall cFO, OR 0.7 [95% CI 0.3, 1.7], *p* = 0.41), renal failure (early cFO, OR 1.3 [95% CI 0.4, 4.1], *p* = 0.69; overall cFO, OR 0.7 [95% CI 0.3, 1.7], *p* = 0.41), or use of CRRT (early cFO, OR 2.3 [95% CI 0.4, 14.0]; overall, cFO, OR 0.5 [95% CI 0.1, 4.9], *p* = 0.59).

**Table 3:** % Fluid overload and mortality

	Early (< 72 h)		Overall (1 week)	
	cFO > 10% vs < 5%		cFO > 10% vs < 5%	
	OR (95% CI)	*P*-value	OR (95% CI)	*P*-value
Univariate				
In-hospital mortality	4.6 (0.7, 30.7)	0.12	3.0 (0.5, 18.7)	0.29
Mortality at 1 year	9.9 (1.8, 54.7)	**0.01**	4.7 (0.8, 24.2)	0.1
Multivariate				
In-hospital mortality	3.3 (0.5, 21.9)	0.22		
Mortality at 1 year	8.6 (1.4, 51.6)	**0.04**		

Bold values indicate statistical significance (*p* ≤ 0.05)

Both early cumulative fluid overload (assessed at 72 h following post-cardiac transplantation) and overall cFO (assessed at 1 week following post-cardiac transplantation) data are presented with respect to the outcome of mortality

Odds of mortality were calculated using a logistic regression model and displayed as odd ratios (OR) with 95% confidence interval (CI)

Multivariable analysis adjusted for age at time of transplant and admission weight for both in-hospital mortality and mortality at 1 year

### Fluid Overload and Late Clinical Outcomes

Early cFO was noted to be associated with an increase in odds of mortality, OR 9.9 (95% CI 1.8, 54.7, *p* = 0.01) at 1 year. This association held true, following a multivariate analysis adjusting for age and weight, OR 8.6 (95% CI 1.4, 51.6, *p* = 0.04) (Table 3). We further assessed whether fluid overload is associated with higher rates of clinical rejection within the first year of transplantation. Although we observed a higher odds risk of overall clinical rejection with early fluid overload (OR 2.0, 95% CI 0.6–6.7; *p* = 0.3), and severe clinical rejection (OR 2.0 95% CI 0.5–9.1; *p* = 0.36), these findings were not statistically significant. This relationship was also not significant with overall cFO compared to those with no cFO. We also found no association between fluid overload and the rate of hospitalizations within the first year of cardiac transplantation (Est 1.6, SE 1.6; *p* = 0.34, by negative binomial regression model).

We also assessed hemodynamic surrogates of primary graft function by evaluating the relationship with fluid overload and cardiopulmonary filling pressures at serial routine time points following cardiac transplantation (Table 4). Although we found early and overall cFO associated with a higher odds risk of elevated right atrial pressure (RAP) > 8 mmHg, OR 2, 95% CI (0.4, 10.8), *p* = 0.42 and OR 1.2, 95% CI (0.3, 4.3), *p* = 0.8, respectively, measured within the first month of transplantation, these results were not statistically significant. Further, overall cFO was associated with a lower odds risk of elevated cardiopulmonary filling pressures, measured as elevated RAP > 8 mmHg, OR 0.1 95%CI (0.01, 0.5), *p* = 0.01 and pulmonary capillary wedge pressure (PCWP) > 12 mmHg, OR 0.1 95%CI (0.01, 0.6), *p* = 0.01 (Table 4), assessed at 3–6 months post-transplant. There was no significant relationship between both early and overall cFO and low cardiac index (CI), as measured by a cardiac index < 2 L/min/m^2^, at all routine time points of cardiac catheterization within the first year of cardiac transplantation.

**Table 4:** % Fluid overload stratification and hemodynamic profiles

	Early (< 72 h)			Overall (1 week)		
	cFO > 10% vs < 5%			cFO > 10% vs < 5%		
	N	OR (95% CI)	*P*-value	*N*	OR (95% CI)	*P*-value
RAP > 8 mmHg						
≤ 1 month	63	2 (0.4, 10.8)	0.42	74	1.2 (0.3, 4.3)	0.8
3–6 months	62	0.5 (0.1, 2.1)	0.33	77	0.1 (0.01, 0.6)	**0.01**
12 months	42	0.9 (0.2, 5.5)	0.93	52	0.3 (0.1, 1.7)	0.17
PCWP > 12 mmHg						
≤ 1 month	62	1.4 (0.3, 7.6)	0.71	73	0.5 (0.1, 1.6)	0.22
3–6 months	62	0.6 (0.1, 2.7)	0.51	77	0.1 (0.01, 0.6)	**0.01**
12 months	42	1.1 (0.2, 6.5)	0.93	52	0.2 (0.02, 1.4)	0.96
CI < 2 L/min/m^2^						
≤ 1 month	63	0.3(0.03, 2.2)	0.21	74	0.5 (0.1, 2.0)	0.32
3–6 months	61	0.96 (0.1, 9.1)	0.51	76	1.0 (0.2, 4.0)	0.96
12 months	42	2.3 (0.2, 28.9)	0.52	52	0.9 (0.03, 24.7)	0.93

Bold values indicate statistical significance (*p* ≤ 0.05)

Both early cumulative fluid overload (assessed at 72 h following post-cardiac transplantation) and overall cFO (assessed at 1 week following post-cardiac transplantation) data are presented with respect to cardiopulmonary filling pressures and cardiac index at respective time points post-cardiac transplantation

Odds of elevated cardiopulmonary filling pressure or low cardiac index were calculated using a logistic regression model and displayed as odd ratios (OR) with 95% confidence interval (CI)

*RAP* right atrial pressure, *PCWP* pulmonary capillary wedge pressure, *CI* cardiac index

## Discussion

The primary results of this study can be summarized as follows. First, consistent with a recent pediatric heart transplant study [21], the incidence of postoperative fluid overload was noted to be higher than what has been previously reported for non-transplant cardiac surgery patients at 23% [13, 30]. Younger age, lower weight, and presence of immediate graft failure needing the use of postoperative ECMO are significant risk factors for both early and overall cumulative postoperative fluid overload. In addition, the presence of cyanotic heart disease was a significant risk factor for early fluid overload. There was no significant relationship between postoperative cFO and immediate post-transplant outcomes. However, at 1-year, early cFO was significantly associated with post-transplant mortality. This relationship was independent of age and weight with a logistic regressions model.

Our findings are consistent with previous data on early volume overload and poor outcomes with pediatric cardiac surgery and post-solid organ transplant [13, 21, 25, 31, 32]. Early fluid overload within 72 h of pediatric cardiac surgery is a known independent predictor of in-hospital mortality and poor cardiac function marked with low cardiac output syndrome [13]. However, we are to our knowledge the first study to show the association of early fluid overload with poor long-term clinical outcomes at 1-year in pediatric heart transplant recipients, suggesting a possible pathophysiologic relationship that extends beyond the immediate perioperative period. ICU-related studies have shown an independent relationship between cumulative fluid balance and long-term mortality among critically ill patients [33, 34]. Mechanistic studies have suggested prolonged fluid overload can lead to direct adverse effects that are inclusive of microvascular injury associated with tissue edema, endothelial dysfunction and shedding of the endothelial glycocalyx, and dysregulated immunity [4, 35–37]. Early perioperative fluid overload is also a known risk factor for early graft failure in a wide spectrum of solid organ transplants [31, 32, 38] and well described, poor predictor of long-term survival in allogeneic hematopoietic stem cell transplantation due to multi-end organ failure [39, 40]. In addition, heart transplant recipients are known to be especially vulnerable to the risk of perioperative volume overload due to abnormal cardiorenal neuroendocrine reflexes from cardiac denervation of the transplanted heart [23, 24]. Further, this abnormal neuroendocrine response in OHT recipients has been found to be positively associated with postoperative volume overload, post-transplant systemic hypertension, and transient early allograft dysfunction in adult studies [22–24]. This may in part suggest a biological plausibility to the association we observed between early cFO and death at 1 year among pediatric heart transplant recipients.

When further examining the relationship of volume overload with mortality, it is important to note that although not statistically significant, there was a trend toward a positive association between rates of empiric clinical rejection within the first year of transplant and early volume overload that was not observed with cumulative volume overload. Our study may have been statistically underpowered to assess this relationship. Further, a significant proportion of pediatric heart transplant recipients with primary allograft dysfunction are known to present as sudden death [41], and there may be an important relationship between early volume overload and allograft survival that are limited by our primary clinical outcomes of measure. Determinants of allograft dysfunction with routine cardiac catheterization are even also limited in assessment, as they lack longitudinal trajectory, prone to positive selection, and often further exclude small children and infants that are limited in size and vascular access for frequent surveillance post-transplant cardiac catheterization protocols. This study did not demonstrate an association with early fluid overload and elevated filling pressures at early selected time points of cardiac catheterization despite prior adult OHT demonstrating this association [22], possibly due to these study limitations listed above. The association between early fluid overload and poor late outcomes may also be driven by proposed mechanisms of surrounding tissue and endothelial damage, leading not only to primary allograft failure but multi-system end-organ damage as well.

This study also confirms that early volume overload among pediatric OHT patient is highly prevalent, with those at greatest risk being those smaller in size and younger. Irrespective if truly rooted by a pathobiological effect or simply a mere epiphenomenon, early postoperative volume overload may represent an early marker of post-transplant death within the first year of OHT transplantation and this finding warrants further investigation. Our study highlights the question whether closer surveillance of those that sustained early volume overload is indicated and if traditional bedside practices, especially among those that are younger and with low body weight, are sensitive to accurate postoperative volume assessment.

Limitations to our study include we did not assess probable or confirmed causes of death in our study as it relates to volume overload. In addition, other early postoperative outcomes such as duration of mechanical ventilation, duration of inotropic support, intensive care unit length of stay were not evaluated in this study, as we felt at the time they were often influenced by a multitude of other factors, not simply by fluid status. In addition, this was a single-center retrospective cohort study and validation of our findings among a larger multi-center, prospective cohort is warranted. The small sample size of our study limited statistical power and increased probability of type ii error with respect to the clinical outcomes of measure and our multivariate analysis. While we employed statistical analyses to adjust for known predictors of poor outcomes in the transplant population such as age and weight, the small retrospective nature of this study cannot fully account for potential confounders that can influence the statistical associations found in this study. Although our results are further supported by a growing body of literature suggesting a pathobiological role of postoperative volume overload and organ dysfunction in both the cardiac surgical and post-transplant population, any causal inferences made with volume overload and late-transplant outcomes in this study is conjectural and cannot be fully understood with this study alone. In addition, the pediatric heart transplant population is a heterogenous population compared to adult OHT recipients, further complicating the interpretation of our results and with data limited in assessing significant test interactions among subgroups.

This study however has several strengths. Among sparse literature, to date, this is the largest study relating postoperative volume overload and clinical outcomes among pediatric heart transplant recipients. It is the first study to assess the relationship with early volume overload and both clinical outcomes and surrogate markers of allograft dysfunction, extended to the first year of transplant. We assessed volume overload using validated cutoff values of volume overload as reported in the literature and further assessed volume overload at two time points in the postoperative period. Finally, we assessed all clinical and surrogate outcomes to standardized criteria parameters for data collection and outcome reporting.

In conclusion, early postoperative volume overload is highly prevalent and correlates with an increased risk of post-transplant mortality among pediatric heart transplant recipients. Our study indicates the relationship between early postoperative volume overload and mortality is beyond the perioperative period and may be an early predictor of mortality at 1-year following transplantation. Larger prospective studies are warranted to better explore this relationship and further evaluate if changes to goal direct fluid therapy in the postoperative period is needed among this vulnerable population.

## Data Availability

ML and MF had full access to all of the data in the study and take responsibility for the integrity of the data and accuracy of the data analysis.

## Abbreviations

FO: Fluid overload
OHT: Orthotopic heart transplant
CPB: Cardiopulmonary bypass time
VIS: Vasoactive inotrope score
Cfo: Cumulative fluid overload
AKI: Acute kidney injury
CRRT: Continuous renal replacement therapy
KDIGO: Kidney Disease Improving Global Outcomes
ISHLT: International Society for Heart and Lung Transplantation
ACR: Acute cellular rejection
AMR: Antibody-mediated rejection
ECLS: Extracorporeal life support
VAD: Ventricular assist device
RAP: Right atrial pressure
PCWP: Pulmonary capillary wedge pressure
CI: Cardiac index
ICU: Intensive care unit
